# Decreased AIRE and promiscuous gene expression in thymus from Down syndrome individuals may explain predisposition to autoimmunity

**DOI:** 10.1186/1479-5876-10-S3-P5

**Published:** 2012-11-28

**Authors:** Roger Colobran, Maria del Pilar Armengol, Eduard Porta, Paula A Correa, Ricardo Pujol-Borrell

**Affiliations:** 1 Immunology Division, Hospital Universitari Vall d’Hebron (HUVH). Vall d’Hebron Research Institute (VHIR), Barcelona, Spain; 2 Immunology - LIRAD, Banc de Sang i Teixits (BST), Institut d’Investigació en Ciències de la Salut Germans Trias i Pujol (IGTP), Badalona, Spain; 3 Dept. of Cell Biology, Physiology and Immunology. Universitat Autònoma de Barcelona (UAB), Bellaterra, Spain

## 

Down syndrome (DS), a chromosomal condition caused by the trisomy of chromosome 21, is associated with various immunological impairments, including a high incidence of autoimmune diseases (e.g., hypothyroidism, type 1 diabetes) and anatomical changes in the thymus. The autoimmune regulator (AIRE) is a transcription factor whose gene maps in 21q22.3 and controls the ectopic expression of a large set of peripheral tissue antigen genes (PTA) in medullary thymic epithelial cells (mTECs). This phenomenon, termed promiscuous gene expression (pGE), plays a key role in central tolerance as demonstrated by the inactivation of AIRE resulting in the rare recessive autoimmune polyendocrinophathy-candidiasis-ectodermal dystrophy syndrome (APECED). DS patients carry three copies of AIRE which may result in its overexpression or, more rarely, its underexpression. To investigate the possibility that DS associated autoimmunity is favored by the impairment of pGE as a consequence of changes in AIRE expression and/or anatomical disorganisation of the thymus, we investigated AIRE, PTA and cell marker gene expression in thymi of DS individuals vs controls. Gene expression was assessed on cDNA from 19 DS and 21 controls total thymus samples by qPCR using TaqMan probes. Interestingly, AIRE gene expression was significantly reduced in thymus from DS vs controls (p = 0.0003). 7 PTA genes (CHRNA1, GAD1, PLP1, KLK3, SAG, TG and TSHR) were also reduced, more markedly for KLK3 and SAG (P = 0.002 and 0.0004 respectively). This changes seems unlikely to result from a reduction in the number of epithelial thymic cell since the levels of keratin expression were not reduced. Allele-specific quantification of three alleles of the AIRE gene demonstrated that, in spite of its overall reduction, the three copies of AIRE genes are expressed in the thymus of DS patients, thus indicating that reduction of AIRE expression is not caused by allele silencing (imprinting). Conclusion, autoimmunity associated to DS is probably in part caused by a reduction of AIRE expression that results in an impaired pGE rather that by a reduction of thymic epithelial cells as part of anatomical disorganization.

